# Prevalence and Factors Associated with Dentin Hypersensitivity among Adult Patients Attending a University Dental Clinic in Trinidad, West Indies. A Cross-Sectional Study

**DOI:** 10.3290/j.ohpd.b871073

**Published:** 2020-12-14

**Authors:** Reisha Rafeek, Rahul Naidu, Tamika Peters, Gina Paul, Vrijesh Tripathi

**Affiliations:** a Senior Lecturer, School of Dentistry, Faculty of Medical Sciences, The University of the West Indies, St. Augustine, Trinidad & Tobago. Idea, reviewed the final paper.; b Professor, School of Dentistry, Faculty of Medical Sciences, The University of the West Indies, St. Augustine, Trinidad & Tobago. Idea, reviewed the final paper.; c Dentist, School of Dentistry, Faculty of Medical Sciences, The University of the West Indies, St. Augustine, Trinidad & Tobago. Collected the data, reviewed the final paper.; d Senior Lecturer, Faculty of Science and Technology, The University of the West Indies, St. Augustine, Trinidad & Tobago. Analysed the data, contributed to the results and discussion, reviewed the final paper.

**Keywords:** dentin hypersensitivity, pain, prevalence

## Abstract

**Purpose:**

To assess the prevalence of dentin hypersensitivity (DH) in an adult population and explore its association with sociodemographic variables, dietary habits and oral health behaviours as there is very little data on this from the Caribbean.

**Materials and Methods:**

A cross-sectional survey was conducted of consecutive dental patients (18 years and over) attending the UWI School of Dentistry polyclinic. Following informed consent, dental examinations were performed and the presence of DH was assessed clinically by sensitivity to an air blast on individual teeth. Patients were also given a questionnaire. Their answers were processed using statistical software (SPSS version 24). Associations between variables were analysed using the chi-squared test.

**Results:**

300 patients participated, with an age range of 18–81 years and a mean age of 44.7 years (SD 15.7). 68.8% were female and the main ethnic groups were African (45.8%), Indian (29.8%) and mixed (24.4%). Over half of the participants (54.9%) reported a history of sensitive teeth and 52% reported sensitivity to the air syringe on one or more teeth. Based on multivariate logistic regression, a history of sensitive teeth was significantly associated with Indo-Trinidadian ethnicity (OR 2.24, 95% CI: 1.23, 4.45), a history of tooth grinding or jaw clenching (OR 0.38, 95% CI: 0.19, 0.76) and consumption of citrus fruits one to two times daily (OR 0.22, 95% CI: 0.06, 0.85). Those who experienced vomiting irregularly were more likely (OR 2.31, 95% CI:

0.96, 5.54) to have DH.

**Conclusion:**

Prevalence of dentin hypersensitivity was high among this sample of dental patients and was associated with ethnicity, tooth grinding and dietary practices.

Dentin hypersensitivity (DH) is characterised by a short, sharp pain arising from exposed dentin in response to stimuli, typically thermal, evaporative, tactile, osmotic or chemical, and which cannot be ascribed to any dental defect or pathology.^9^An individual would experience this as an instantaneous, transient pain and discomfort that lasts for a few moments after a stimulus, such as hot or cold fluid, has been removed.^5^

DH can be diagnosed by a patient’s self-report of pain and the exclusion of other conditions which may cause sensitivity, such as dental caries, fractured teeth, marginal leakage of restorations and pulpitis. This sensitivity arises due to loss of enamel or gingival recession and the exposure of dentinal tubule openings to the oral environment. This exposure causes fluid movement in the tubules, which activates nerve endings in the pulp/dentin interface andthus elicits a pain response.^7^

The international prevalence of DH varies: Europe 41.9%,^34^ USA 12.3%,^13^ Jordan 66.4%,^2^ India (Punjab) 25.0%,^14^ Brazil 33.4%,^10^ China 33.7%,^20^ Africa 52.8%.^4^ This wide range can be explained by several factors, such as sampling frames (regions, country, cities), general or specialist practice location,^31^ diagnostic criteria and whether the evaluation is based on patient-based questionnaires^3^,^18^ or clinical evaluation.^15^,^16^,^31^

Risk factors associated with DH are varied, and include periodontal disease and its treatment,^32^ tooth wear,^1^ toothbrushing habits,^19^ diet (e.g. acidic food and drink),^22^,^23^ and gastric reflux.^6^

Trinidad and Tobago is a two-island state, the most southerly of the Caribbean chain of islands near the coast of Venezuela, with a population of 1.3 million. Due to its colonial history as a former British territory in the West Indies, Trinidad and Tobago has a diverse ethnic composition, with the main ethnic groups being Indo-Trinidadian (35.4%), Afro-Trinidadian 34.2% and mixed ethnicity (23%).^24^

There is very little data on the prevalence of DH in the Caribbean, although in Trinidad tooth wear was found to be common among 72%,^26^ and in another study, non-carious cervical lesions sensitive to air were found in 45% of cases.^29^

The aim of this study was to assess the prevalence of dentin hypersensitivity in an adult Caribbean population and explore associations with demographic variables, oral health behaviours and dietary practice. 

## MATERIALS AND METHODS

The study involved a cross-sectional survey of consecutive patients over 18 years old attending a university dental polyclinic in Trinidad for dental care. The University of the West Indies School of Dentistry polyclinic is a teaching clinic providing general or specialist care for adult dental patients. 

Ethical approval for this research was obtained from the University of the West Indies Campus Research Ethics Committee (CEC198/05/16) and individual written informed consent was obtained for participation in the study. 

Patients who consented to participate were asked to fill out a questionnaire and underwent a dental examination. Examinations were undertaken by two dentists trained in the use of the assessment criteria which were agreed upon by the two participating dentists. During the period of data collection, September 2016 to May 2017, a sample size of 300 dental patients was achieved. 

### Sample Selection and Size 

Patients attended the dental polyclinic by appointment following self-referral or referral from a private practice or health center.

A power calculation was not undertaken. A sample size of 300 patients was considered achievable during the period of data collection and amenable to statistical analysis.

### Inclusion and Exclusion Criteria 

Patients of the dental polyclinic were invited to participate in the study based on the following criteria: Patients over the age of 18 years attending for their first appointment at the dental clinic were included. Exclusion criteria: orthodontic appliances, any disease requiring analgesics drugs, tranquilizers or mood-altering medication.

### Questionnaire

The questionnaire included the variables of age, gender, occupation, gastrointestinal symptoms, dental history, oral health behaviours and dietary practices. The questionnaire is presented in Table 1.

**Table 1:** Dentin hypersensitivity questionnaire

	Please fill in or circle only one option for each question					
Age	_____ years					
Sex	Male	Female				
Ethnic descent	African	Indian	Chinese	Caucasian	Mixed	Other
Occupation	Housewife	Student	Employed	Unemployed	Retired	
How often do you experience heartburn?	Not at all	Irregularly	Daily	Weekly		
How often do you experience gastric reflux?	Not at all	Irregularly	Daily	Weekly		
How often do you experience vomiting?	Not at all	Irregularly	Daily	Weekly		
Are you aware of grinding /clenching your teeth?	Yes	No				
Have you ever had a mouthguard/splint made?	Yes	No				
Do you have sensitive teeth?	Yes	No				
Are you vegetarian?	Yes	No				
How often do you brush your teeth?	Once a day	Twice a day	More than twice daily			
What type of toothbrush do you use?	Soft	Medium	Hard	Don’t know		
How often do you consume any of the following:						
Citrus fruits	Never	1 – 3 times per month	1 – 3 times per week	1 – 2 times per day	More than twice daily	
Fruit juice	Never	1 – 3 times per month	1 – 3 times per week	1 – 2 times per day	More than twice daily	
Soft drinks	Never	1 – 3 times per month	1 – 3 times per week	1 – 2 times per day	More than twice daily	
Sports drinks	Never	1 – 3 times per month	1 – 3 times per week	1 – 2 times per day	More than twice daily	
Alcohol	Never	1 – 3 times per month	1 – 3 times per week	1 – 2 times per day	More than twice daily	
Chewing gum	Never	1 – 3 times per month	1 – 3 times per week	1 – 2 times per day	More than twice daily	
Mints	Never	1 – 3 times per month	1 – 3 times per week	1 – 2 times per day	More than t wice daily	
Effervescent vitamin C	Never	1 – 3 times per month	1 – 3 times per week	1 – 2 times per day	More than twice daily	

### Diagnosis of Hypersensitivity

The diagnosis of hypersensitivity was made by a blast of air from a 3-way dental syringe for 1 s from a distance of 1 cm from the tooth surface, including the buccal/labial, occlusal and lingual/palatal surface. Adjacent teeth were protected from the air blast by the examiner’s fingers, and the reaction was noted. 

Teeth with any of the following were excluded: root canal treatment, crowned teeth, abutment teeth for denture or bridge, teeth with marginal restorations interfering with DH evaluation, teeth with buccal (facial) restorations.

A 10-point visual analogue scale (VAS) was presented to the patient to quantify their pain response to the air blast. This linear 10-digit scale was marked from 0–1 (no pain), 2–4 (mild pain), 5–7 (moderate pain) and 8–10 (severe pain). The patient asked to report VAS score immediately after the air blast.

Recession was recorded to determine extent of exposed dentin and the relationship with sensitivity to the air-blast, across the dentition. The presence of recession was recorded using a graduated periodontal probe (UNC 15, Hu-Friedy; Chicago, IL, USA) and this was measured from cementoenamel junction to the apical gingival margin.

### Statistical Analysis

Data processing and analysis was conducted using SPSS version 24 (IBM; Armonk, NY, USA) and Stata version 14.1 (Stata; College Station, TX, USA). This included descriptive statistics and analysis to explore associations between DH and questionnaire variables, using univariate and multivariate logistic regression. For our multivariate analyses, we used stepwise logistic regression, modeling those covariates that were of clinical interest and statistically significant at (p ≤ 0.20) in univariate analyses.

### RESULTS

### Descriptive Characteristics

The study was conducted on 300 patients. Information on DH was missing in five patients and hence, for purposes of analyses, data on 295 patients were analyzed. The age range of participants was 18–81 years with a mean of 44.7 years. 69% were female. Age was grouped into five categories: ≤35, 36–45, 46–55, 56–65, >65. 35.2% of the patients were ≤35 years of age while 11.8% were over 65 years of age at the time of presentation at the clinic. The main ethnic groups were African 45.8%, Indian 29.8% and mixed 24.4%. Nearly 48% were currently employed (Table 2). 

**Table 2:** Crude prevalence and logistic regression for history of sensitive teeth

Variables	Sensitive teeth		Total (295)	Crude prevalence (95%CI)	Univariate OR (95% CI)	Multivariate OR (95% CI)
	No n = 133 (%)	Yes n = 162 (%)				
**Age (years)**						
≤35	46 (34.6)	58 (35.8)	104 (35.2)	0.56 (0.46, 0.65)	1	
36–45	22 (16.5)	31 (19.1)	53 (18.0)	0.58 (0.45, 0.71)	1.12 (0.57, 2.18)	
46–55	21 (15.8)	32 (19.8)	53 (18.0)	0.60 (0.47, 0.73)	1.21 (0.62, 2.36)	
56–65	22 (16.5)	28 (17.3)	50 (17.0)	0.56 (0.42, 0.69)	1.01 (0.51, 1.99)	
>65	22 (16.5)	13 (8.0)	35 (11.8)	0.37 (0.22, 0.54)	0.47 (0.21, 1.02)	
Age (18–81 years)	45.7±16.9	43.9±14.8	44.7±15.7			
**Sex**						
Male	51 (39.1)	40 (24.7)	92 (31.2)	0.43 (0.34, 0.54)	1	
Female	81 (60.9)	122 (75.3)	203 (68.8)	0.60 (0.53, 0.67)	1.96 (1.19, 3.22)**	
Ethnicity						
Afro-Trinidadian	65 (48.9)	70 (43.2)	135 (45.8)	0.52 (0.43, 0.60)	1	1
Indo-Trinidadian	27 (20.3)	61 (37.7)	88 (29.8)	0.69 (0.59, 0.78)	2.10 (1.19, 3.69)*	2.24 (1.23, 4.45)
Mixed and other	41 (30.8)	31 (19.1)	72 (24.4)	0.43 (0.32, 0.55)	0.70 (0.40, 1.25)	0.61 (0.30, 1.25)
**Occupation **						
Housewife	19 (14.3)	31 (19.1)	50 (16.9)	0.62 (0.48, 0.74)	1	
Student	17 (12.8)	21 (13.0)	38 (12.9)	0.55 (0.39, 0.70)	0.76 (0.32, 1.78)	
Employed	67 (50.4)	73 (45.1)	140 (47.5)	0.52 (0.44, 0.60)	0.67 (0.35, 1.29)	
Unemployed	6 (4.5)	15 (9.3)	21 (7.1)	0.71 (0.49, 0.87)	1.53 (0.51, 4.63)	
Retired	24 (18.0)	22 (13.6)	46 (15.6)	0.47 (0.34, 0.62)	0.56 (0.25, 1.27)	

*p<0.05, **p<0.01.

Over one-quarter (26.7%) had a history of grinding or clenching their teeth. The majority (68.9%) reported twice daily toothbrushing. With respect to gastro-intestinal symptoms, heartburn, reflux, or vomiting was experienced irregularly by 20.5%, 23.6% and 14.2% of the sample, respectively (Table 3).

**Table 3:** Crude prevalence and logistic regression for history of sensitive teeth (cont.)

How often do you experience any of the following medical conditions?	Sensitive teeth		Total	Crude prevalence (95% CI)	Univariate OR (95% CI)	Multivariate OR (95% CI)
	No n = 133 (%)	Yes n = 162 (%)				
**Heartburn**						
Not at all	94 (75.2)	108 (70.6)	202 (72.7)	0.53 (0.47, 0.60)	1	
Irregularly	23 (18.4)	34 (22.2)	57 (20.5)	0.60 (0.46, 0.72)	1.29 (0.71, 2.34)	
Daily/weekly	8 (6.4)	11 (7.2)	19 (6.8)	0.58 (0.35, 0.78)	1.20 (0.46, 3.10)	
**Gastric reflux**						
Not at all	94 (74.6)	96 (62.3)	190 (67.9)	0.51 (0.43, 0.58)	1	
Irregularly	24 (19.0)	42 (27.3)	66 (23.6)	0.64 (0.51, 0.74)	1.71 (0.96, 3.05)	
Daily/weekly	8 (6.3)	16 (10.4)	24 (8.6)	0.67 (0.46, 0.83)	1.96 (0.80, 4.79)	
**Vomiting **						
Not at all	108 (89.3)	121 (82.9)	229 (85.8)	0.53 (0.46, 0.59)	1	1
Irregularly	13 (10.7)	25 (17.1)	38 (14.2)	0.66 (0.49, 0.79)	1.72 (0.84, 3.52)	2.31 (0.96, 5.54)
**Do you grind or clench?**						
Yes	25 (18.9)	52 (33.3)	77 (26.7)	0.68 (0.56, 0.77)	1	1
No	107 (81.1)	104 (66.7)	211 (73.3)	0.49 (0.42, 0.56)	0.47 (0.27, 0.81)**	0.38 (0.19, 0.76)**
**Have you ever had a mouth-guard or splint?**						
Yes	7 (5.3)	7 (4.5)	14 (4.9)	0.50 (0.25, 0.74)	1	
No	124 (94.7)	148 (95.5)	272 (95.1)	0.54 (0.48, 0.60)	1.19 (0.41, 3.50)	
**Are you vegetarian?**						
Yes	12 (9.0)	19 (11.8)	31 (10.5)	0.61 (0.43, 0.77)	1	
No	121 (91.0)	142 (88.2)	263 (89.5)	0.53 (0.47, 0.60)	0.74 (0.35, 1.59)	
**How often do you brush your teeth per day?**						
Once	23 (17.4)	20 (12.3)	43 (14.6)	0.47 (0.32, 0.61)	1	
Twice	82 (62.1)	120 (74.1)	202 (68.7)	0.59 (0.52, 0.66)	1.68 (0.87, 3.26)	
More than twice	27 (20.5)	22 (13.6)	49 (16.7)	0.45 (0.31, 0.56)	0.94 (0.41, 2.13)	
**What type of brush do you use?**						
Soft	34 (26.0)	47 (29.0)	81 (27.6)	0.58 (0.47, 0.68)	1	
Medium	80 (61.1)	95 (58.6)	175 (59.7)	0.54 (0.47, 0.62)	0.86 (0.51, 1.46)	
Hard	17 (13.0)	20 (12.3)	37 (12.6)	0.54 (0.37, 0.69)	0.85 (0.39, 1.86)	

**p<0.01.

Just under one-third consumed sports drinks 1–3 times per month. 10% consumed soft drinks more than twice daily. One-third of participants consumed alcohol 1–3 times per month (Table 4).

**Table 4:** Crude prevalence and logistic regression for history of sensitive teeth (cont.)

	Sensitive teeth		Total	Crude prevalence (95% CI)	Univariate OR (95% CI)	Multivariate OR (95% CI)
	No n = 133 (%)	Yes n = 162 (%)				
**How often do you consume citrus fruit?**						
Never	9 (6.9)	15 (9.6)	24 (8.4)	0.63 (0.41, 0.80)	1	
1–3 times per month	34 (26.2)	50 (31.8)	84 (29.3)	0.60 (0.49, 0.70)	0.88 (0.35, 2.25)	0.78 (0.26, 2.29)
1–3 times per week	56 (43.1)	76 (48.4)	132 (46.0)	0.58 (0.49, 0.66)	0.82 (0.33, 1.99)	1.16 (0.41, 3.27)
1–2 times per day	22 (16.9)	12 (7.6)	34 (11.8)	0.35 (0.21, 0.53)	0.33 (0.11, 0.97)*	0.22 (0.06, 0.85)*
More than twice daily	9 (6.9)	4 (2.5)	13 (4.5)	0.31 (0.11, 0.60)	0.27 (0.06, 1.12)	0.36 (0.08, 1.70)
**How often do you consume citrus fruit juice?**						
Never	13 (10.4)	18 (11.3)	31 (10.9)	0.58 (0.40, 0.74)	1	
1–3 times per month	24 (19.2)	33 (20.6)	57 (20.0)	0.58 (0.45, 0.70)	0.99 (0.41, 2.41)	
1–3 times per week	44 (35.2)	74 (46.3)	118 (41.1)	0.63 (0.53, 0.71)	1.22 (0.54, 2.72)	
1–2 times per day	38 (30.4)	26 (16.3)	64 (22.5)	0.41 (0.29, 0.53)	0.49 (0.21, 1.18)	
More than twice daily	6 (4.8)	9 (5.6)	15 (5.3)	0.60 (0.33, 0.81)	1.08 (0.31, 3.80)	
**How often do you consume softdrinks?**						
Never	32 (25.2)	38 (24.4)	70 (24.7)	0.54 (0.42, 0.66)	1	
1–3 times per month	35 (27.6)	47 (30.1)	82 (29.0)	0.57 (0.46, 0.68)	1.13 (0.60, 2.15)	
1–3 times per week	32 (25.2)	33 (21.2)	65 (23.0)	0.51 (0.37, 0.63)	0.87 (0.44, 1.71)	
1–2 times per day	16 (12.6)	21 (13.5)	37 (13.1)	0.57 (0.40, 0.71)	1.11 (0.50, 2.47)	
More than twice daily	12 (9.4)	17 (10.9)	29 (10.2)	0.59 (0.40, 0.75)	1.19 (0.50, 2.86)	
**How often do you consume sport drinks?**						
Never	63 (50.4)	82 (52.2)	145 (51.4)	0.57 (0.48, 0.64)	1	
1–3 times per month	40 (32.0)	50 (31.8)	90 (31.9)	0.56 (0.45, 0.66)	0.96 (0.57, 1.63)	
1–3 times per week	17 (13.6)	20 (12.7)	37 (13.1)	0.54 (0.37, 0.69)	0.90 (0.44, 1.87)	
1–2 times per day or more	5 (4.0)	5 (3.2)	10 (3.5)	0.50 (0.21, 0.78)	0.77 (0.21, 2.77)	
**How often do you consume alcohol?**						
Never	68 (53.1)	86 (54.8)	154 (54.0)	0.56 (0.48, 0.64)	1	
1–3 times per month	39 (30.5)	56 (35.7)	95 (33.3)	0.56 (0.48, 0.68)	1.14 (0.68, 1.91)	
1–3 times/ week or more	21 (16.4)	15 (9.6)	36 (12.6)	0.42 (0.27, 0.58)	0.57 (0.27, 1.18)	
**How often do you use chewing gum?**						
Never	61 (48.8)	63 (39.4)	124 (43.5)	0.51 (0.42, 0.60)	1	1
1–3 times per month	35 (28.0)	57 (35.6)	92 (32.3)	0.62 (0.51, 0.71)	1.58 (0.91, 2.73)	1.46 (0.74, 2.88)
1–3 times per week	23 (18.4)	24 (15.0)	47 (16.5)	0.51 (0.37, 0.65)	1.01 (0.52, 1.98)	0.50 (0.21, 1.14)
1–2 times per day or more	6 (4.8)	16 (10.0)	22 (7.7)	0.73 (0.50, 0.87)	2.58 (0.95, 7.03)	2.27 (0.69, 7.52)
**How often do you consume mints?**						
Never	21 (16.4)	29 (18.1)	50 (17.4)	0.58 (0.44, 0.71)	1	
1–3 times per month	44 (34.4)	49 (30.6)	93 (32.3)	0.53 (0.42, 0.63)	0.81 (0.40, 1.61)	
1–3 times per week	47 (36.7)	52 (32.5)	99 (34.4)	0.53 (0.43, 0.82)	0.80 (0.40, 1.59)	
1–2 times per day	10 (7.8)	21 (13.1)	31 (10.8)	0.68 (0.49, 0.81)	1.52 (0.59, 3.89)	
More than twice daily	6 (4.7)	9 (5.6)	15 (5.2)	0.60 (0.34, 0.81)	1.09 (0.34, 3.52)	
**How often do you consume effervescent vitamin C?**						
Never	59 (48.0)	76 (49.0)	135 (48.6)	0.56 (0.48, 0.64)	1	
1–3 times per month	34 (27.6)	46 (29.7)	80 (28.8)	0.58 (0.46, 0.68)	1.05 (0.60, 1.84)	
1–3 times per week	13 (10.6)	23 (14.8)	36 (12.9)	0.64 (0.47, 0.79)	1.37 (0.64, 2.94)	
1–2 times per day or more	17 (13.8)	10 (6.5)	27 (9.7)	0.37 (0.20, 0.57)	0.46 (0.20, 1.07)	

*p<0.05.

### History of Dentin Hypersensitivity

Based on the questionnaire, over half the participants (54.5%) had a history of sensitive teeth. 

More than half of the patients in each of the age categories had DH (varying from 56% to 60%), although DH was found less frequently among those over 65 years of age (37%). Prevalence was higher among females (60%), Indo-Trinidadians (69%), unemployed (71%) and housewives (62%). Among medical conditions, prevalence was higher among those experiencing gastric reflux irregularly (64%) and on a daily/weekly basis (67%), vomiting irregularly (66%), grinding or clenching teeth (68%). In terms of dietary habits, prevalence was lower among those consuming citrus fruit more than twice daily (31%) and once or twice a day (35%), but was higher among those chewing gum once to twice or more per day (73%). 

### Sensitivity to Air Blast and Severity of Discomfort

**Fig 1:**
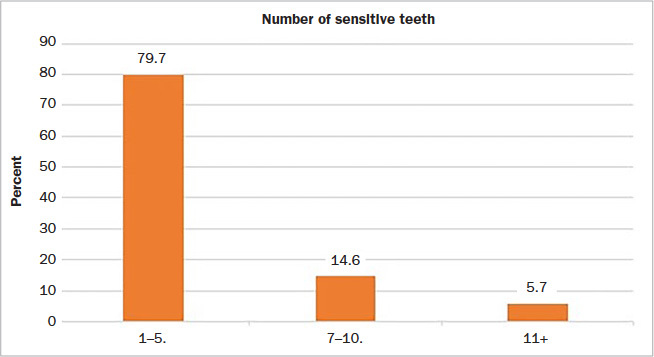
Proportion of participants with teeth sensitive to air blast (N=300).

**Fig 2:**
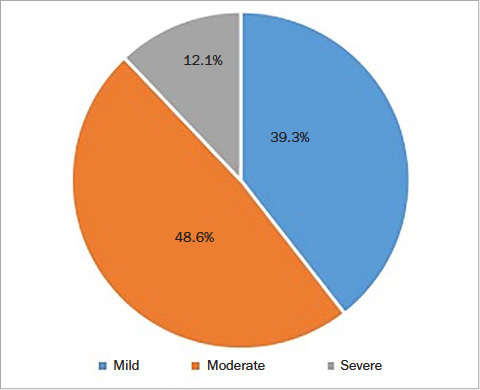
Severity of pain upon air blast based on the VAS.

Fifty-two percent of participants reported sensitivity to the air-blast test, which was the clinical measure of dentin hypersensitivity in this study. Among those who were sensitive to the air blast, for the majority (79.7%), this was on 1–5 teeth (Fig 1). The sensitivity arose mostly from the posterior teeth and more in premolars than molars. Based on the VAS, where participants rated their actual level of discomfort in relation to the air-blast test, almost half (48.6%) rated this as moderate pain and 12.1% as severe pain (Fig 2).

### Recession

Gingival recession was present among 36% of participants, ranging from 1-24 teeth with 83.3% of this recession detected on 1-5 teeth. Recession also more frequently occurred on the posterior than the anterior teeth and more frequently on premolars than molars. 

### Univariate Analysis 

Our univariate analyses showed that age was a protective factor, that is, as patients aged, they were less likely to have DH than younger patients under 35 years of age. Females were nearly 2 times (OR 1.96, 95% CI: 1.19, 3.22) more likely to have DH than males. Indo-Trinidadians were 2 times (OR 2.1, 95%CI 1.19, 3. 69) more likely to have DH than Afro-Trinidadians. For the mixed population, their mixed ethnicity was a protective factor, but this was not statistically significant. Those who did not grind or clench were 53% less likely to have DH than those who ground or clenched teeth. Eating citrus fruits was a protective factor compared to those who never eat citrus fruits. Eating citrus fruits 1–2 times daily was a statistically significant protective factor, as they were 67% less likely to have DH than those who did not. Chewing gum 1–2 times per day was a risk factor that increased the chances of having DH by 2.5 times (OR 2.58, 95% CI: 0.95, 7.03). Those who experienced vomiting irregularly were 72% more likely (OR 1.72, 95% CI: 0.84, 3.52) to have DH than those who did not and those who experienced gastric reflux daily/weekly were twice as likely to have DH (OR 1.96, 95% CI: 0.80, 4.79) than those not experiencing gastric reflux. However, these were not statistically significant factors. 

### Multivariate Analysis

In the multivariate analysis, Indo-Trinidadians were two times (OR 2.24, 95% CI: 1.23, 4.45) more likely to have DH than Afro-Trinidadians. This was a statistically significant difference. Those who did not grind or clench teeth were 62% less likely to have DH than those who did. Consumption of citrus fruits 1–2 times a day reduced the chance of having DH by 78% compared to those who never eat citrus fruits. Chewing gum and vomiting irregularly were not found to yield statistically significant differences in multivariate analysis. 

## DISCUSSION

Dentin hypersensitivity was common in this sample of adult dental patients with just over half (54.9%) reporting a history of sensitivity or having clinical symptoms to the air-blast test (52%). There is a wide range of prevalence of DH internationally, ranging from 12.3% in the USA, to 13 25% in Punjab, India,^14^ 37.2% in Chandigarh, India,^30^ 33.3% in Brazil,^8^ 33.7% in Xiang City, China,^20^ to 34.5% in multiple provinces in China.^33^ A European study collected data from France, Spain, Italy, UK, Finland, Latvia and Estonia and found that 26.8% reported DH and 41.9% reported experiencing DH in response to cold air stimulation. Within that study, DH prevalence ranged from 30% in Finland 30% to 47% in Italy.^34^ The DH prevalence was reported to be higher (52.8%) in Nigeria, Africa^4^ and even higher (66.4%) in Jordan.^2^ The prevalence in this study was in the higher range reported internationally, and similar to data from Africa and the Middle East.

The differences may be due to the type of settings from which the sample of patients came, such as general practice,^13^,^34^ specialist periodontology clinics,^17^,^27^ and university hospital settings,^21^,^27^,^31^ or from the methods used, such as questionnaires or clinical cold air tests. This study was conducted in a university hospital academic setting; other studies in that setting reported DH prevalences of 44%^21^ and 67.7% to a cold air blast.^27^ Despite the trend of older participants having lower prevalence of DH, a history of DH was not statistically significantly associated with age in the regression analysis. This is in contrast to other studies, where DH and age were statistically significantly associated.^3^,^17^,^21^,^33^

In the univariate analysis, females were twice as likely to have DH. This is in agreement with the common finding in the literature that females were more likely to have DH.^4^,^15^,^16^,^33^

DH was statistically significantly associated with ethnicity. This finding contrasts to research that reported no association with ethnicity.^13^

Along with a culture and heritage consistent with most of the islands of the English-Speaking Caribbean, Trinidad and Tobago also has diverse ethnicity due to its unique colonial history which influences dietary practices. For example, a preference for sour (e.g. preserved mango) and hard snacks (e.g. fried /roasted nuts, chick peas and lentils) is common among the Indo-Trinidadian community. Also, previous findings in a Trinidad population reported grinding and clenching was common and associated with tooth surface loss^26^ and the presence of non-carious cervical lesions,^29^ which can predispose to DH. However, grinding and clenching were not found to be a statistically significant risk factor for DH in this study, which agreed with other studies that found bruxism habits not to be statistically significantly associated with DH.^2^,^13^

Gastric reflux and vomiting were found to be associated with DH in this study, and this is consistent with the findings of other studies.^10^,^34^

Fresh fruit is readily available in the Caribbean and common in the diet. However, the regression analysis showed that the consumption of citrus fruits was associated with a lower chance of DH. This finding was contradictory to the literature, where it is known that dental erosion from citric acid can lead to DH. This surprising finding may also be explained by people in this sample who ate citrus fruit frequently but also used measures to mitigate the acidity. For instance, to suit local taste preferences, fresh citrus fruits are commonly squeezed and the juice heavily sweetened and diluted before consumption. In addtion, the more popular citrus fruits in Trinidad are sweeter varieties such as clementine oranges (locally known as Portugals). Furthermore, it is possible that those who consumed citrus fruits more frequently may have also been using desensitizing toothpaste; however, such data was not collected.

The apparent increased risk of DH among patients who chewed chewing gum more regularly is also unexpected and again not supported by the literature, as chewing gum stimulates saliva production, which is known to be protective against acid erosion and subsequent sensitivity. This finding might be explained by these patients having additional dietary factors or habits related to increased risk of in DH, of which they were unaware and therefore did not report in this research, for instance a grinding or clenching habit at night. 

The VAS indicated that pain from the air blast was rated as moderate or severe in the majority of participants, indicating that DH should be considered a serious problem in this population. The impact of DH on oral health-related quality of life (OHRQoL) therefore requires further investigation. The present study found that the teeth affected most by DH were premolars, followed by molars, which agrees with other studies.^17^,^18^,^21^,^33^ Gingival recession has been generally accepted as a predisposing factor for DH^1^ and was present in over one-third of the subjects in this study.

This present research confirms that protocols for prevention of DH in adult dental patients should include a detailed medical history as well as social and demographic characteristics, taking cultural and dietary practices into account along with evidence-based clinical management strategies.

Clinical management of DH depends on identifying the cause and predisposing factors, then preventing or removing them. The prevention of DH can be self-care strategies, such as ensuring proper toothbrushing techniques with a soft-bristle toothbrush, avoiding abrasive toothpastes, use of desensitizing dentrifices and mouthrinses and reducing the frequency of foods and drinks containing acids. Management of DH involves the interruption of the neural response to pain stimuli and occlusion of the exposed dentin tubules to block the hydrodynamic mechanism of pain.^11^

There is a plethora of research studies investigating the efficacy of desensitizing toothpastes using either potassium salts to interrupt the neural response to pain stimuli or desensitizing toothpastes based on occluding the tubules with a natural of artificial smear layer or depositions of precipitate in the tubules from strontium, stannous and calcium phosphate particles.^11^

Recent advances now include a natural mineral formation, such as Pro-Argin technology, which contains arginine, calcium carbonate and fluoride. This is reported to significantly reduce DH instantly after a single, professional application of the product^31^ and after brushing with it twice daily.^12^ A meta-analysis has indicated that arginine-containing toothpaste is effective against DH.^33^ NovaMin, a bioactive glass, is a calcium sodium phosphosilicate material which reacts with saliva to release sodium. This not only increases the salivary pH but also prompts release of calcium and phosphate ions, which precipitate to form a layer over the exposed dentin, subsequently reducing DH.^25^

These desensitizing dentrifices are only one aspect of DH treatment that is self-administered. Management strategies also include professional application of varnishes, adhesive resin cements and restorative materials. More recent strategies include Heal Ozone, in which ozone penetrates the tubules and allows mineral ingress to seal the tubules.^8^

### Limitations 

The generalizability of the findings in this study are limited due to use of a convenience sample of dental patients in a hospital setting and may not be representative of the general population.

## CONCLUSION 

The prevalence of dentin hypersensitivity was high among this sample of dental patients and associated with ethnicity, tooth grinding and dietary practices. Management of this condition should include dietary advice, modification of oral habits, advice on toothbrushing techniques and use of desensitizing dentrifices.
